# Foreign Body Reaction After Hip Augmentation Surgery: A Case Report

**DOI:** 10.7759/cureus.38337

**Published:** 2023-04-30

**Authors:** Harpreet Grewal, Gurmanpreet K Sidhu

**Affiliations:** 1 Radiology, Florida State University College of Medicine, Pensacola, USA; 2 Pathology, Government Medical College, Patiala, Patiala, IND

**Keywords:** early detection, surgical technique, risk factors prevention strategies, magnetic resonance imaging (mri), hip augmentation cosmetic surgery foreign body reaction (fbr)

## Abstract

Hip augmentation cosmetic surgery is an increasingly popular procedure for patients seeking to enhance their body contour and improve their self-image. Despite its benefits, complications can arise, including the rare but serious foreign body reaction (FBR). We present a case of a 32-year-old patient with a history of hip augmentation cosmetic surgery who presented with persistent hip pain. A comprehensive clinical evaluation, followed by magnetic resonance imaging (MRI), revealed a foreign body reaction associated with the cosmetic augmentation procedure. This case report aims to describe the clinical presentation, diagnosis, and management of FBR in patients who have undergone hip augmentation cosmetic surgery. We will also discuss potential risk factors, prevention strategies, and the importance of early detection and intervention to avoid severe complications and improve patient outcomes. By sharing this case, we aim to raise awareness among healthcare professionals about this rare but significant complication of foreign body reaction and emphasize the need for close monitoring and timely intervention in patients who have undergone hip augmentation cosmetic surgery.

## Introduction

Hip augmentation cosmetic surgery is an increasingly sought-after procedure, as it aims to enhance body contour and improve self-confidence by addressing aesthetic concerns related to the hip area. While this procedure has brought satisfaction to many patients, it is not without risks. One rare but serious complication that can arise is the foreign body reaction (FBR) [1]. FBRs can lead to pain, inflammation, and potentially severe consequences if not diagnosed and managed appropriately. In this case report, we present a 32-year-old patient with a history of hip augmentation cosmetic surgery who developed a foreign body reaction, as identified through magnetic resonance imaging (MRI), following the presentation of persistent hip pain. This report serves to raise awareness among healthcare professionals about this rare complication, emphasizing the importance of early detection, proper patient selection, and preventive measures to minimize risks and improve patient outcomes in those undergoing hip augmentation cosmetic surgery.

## Case presentation

A 32-year-old female patient presented with a chief complaint of persistent hip pain that had worsened over the past few months. The pain was described as dull and constant, with occasional sharp episodes that limited her daily activities and affected her overall quality of life. The patient had no significant past medical history except for a hip augmentation cosmetic surgery performed two years ago at an outpatient facility. The procedure involved the injection of synthetic fillers in both hips (liquid silicone), which initially provided the desired aesthetic outcome, but the pain started to manifest six months after the surgery.

Upon physical examination, the patient's hip area appeared to be mildly swollen without tenderness. The range of motion in the affected hip was slightly reduced due to pain. The detailed clinical examination findings were unavailable since the clinical exam was performed at an outside facility, and the case reports aim to emphasize the MRI findings in patients with foreign body reaction after the hip augmentation procedure. No signs of infection or systemic symptoms such as fever, weight loss, or malaise were reported. Laboratory tests, including a white blood count (5,500 cells/mcL), erythrocyte sedimentation rate (12 mm/hr), and C-reactive protein levels (1.0 mg/L), were within normal limits.

Given the patient's history and clinical presentation, an MRI of the affected hip was performed to further investigate the cause of the pain. The MRI revealed heterogeneous signal intensity within the hips with intramuscular edema, suggestive of a foreign body reaction. No radiological signs of infection, localized granuloma, or other complications were observed. The patient underwent treatment with 25 mg of etanercept, administered subcutaneously on a twice-weekly basis, following the confirmation of negative tuberculin (purified protein derivative) test results. Within two weeks, there was a significant reduction in pain and tenderness experienced by the patient.

## Discussion

Hip augmentation cosmetic surgery is a procedure that involves the use of various implants or soft tissue fillers to enhance the hip contour and improve body appearance. Although the procedure is generally considered safe, it can be associated with a range of complications, including infection, implant migration, tissue necrosis, and, rarely, FBR [2].

**Figure 1:**
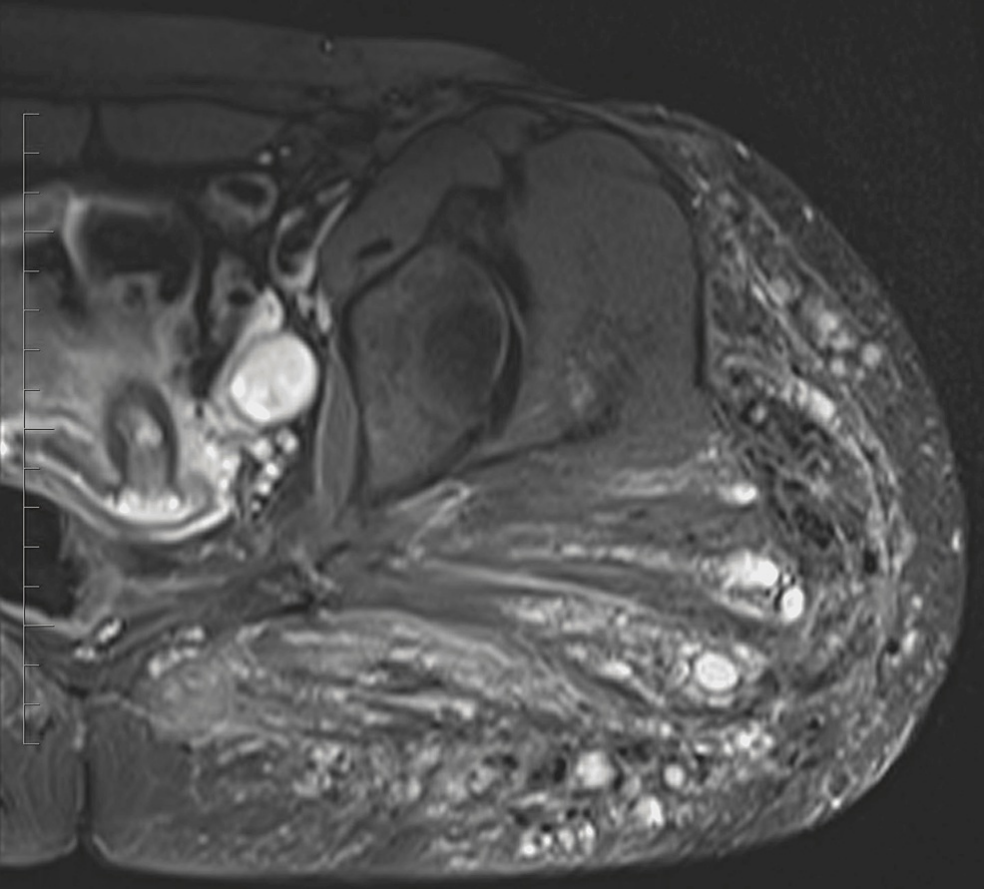
Axial T2 fat-saturated image demonstrates post-procedural changes from prior hip augmentation surgery. There are intramuscular cystic changes with heterogeneously signal intensity.

**Figure 2:**
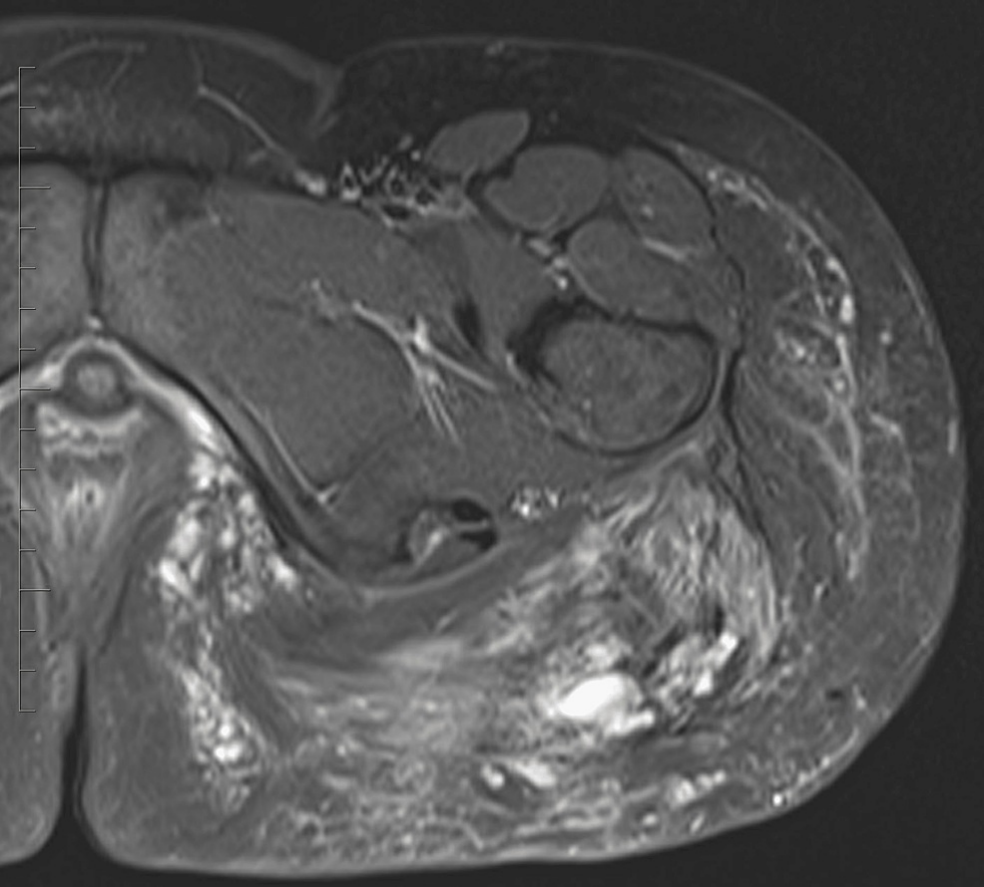
Axial fat-saturated T2 weighted image demonstrates intramuscular edema within the left gluteus musculature.

**Figure 3:**
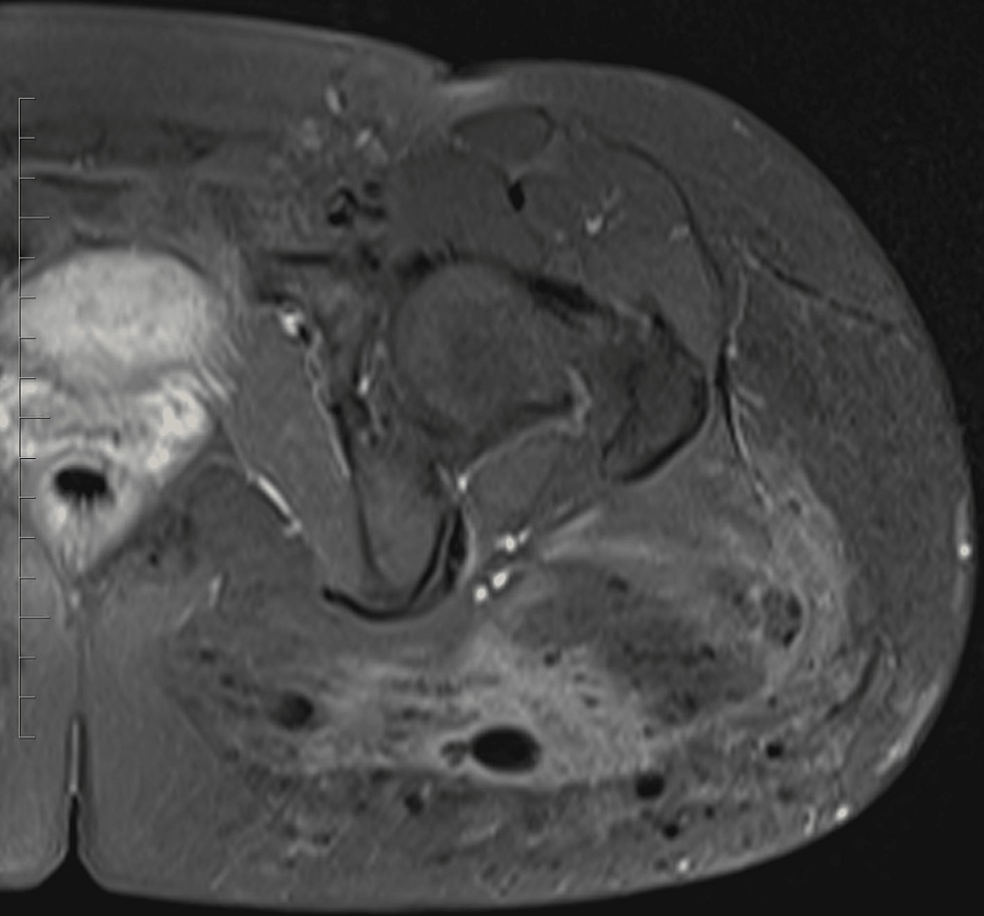
Axial T1 fat-saturated image shows cystic intramuscular changes.

**Figure 4:**
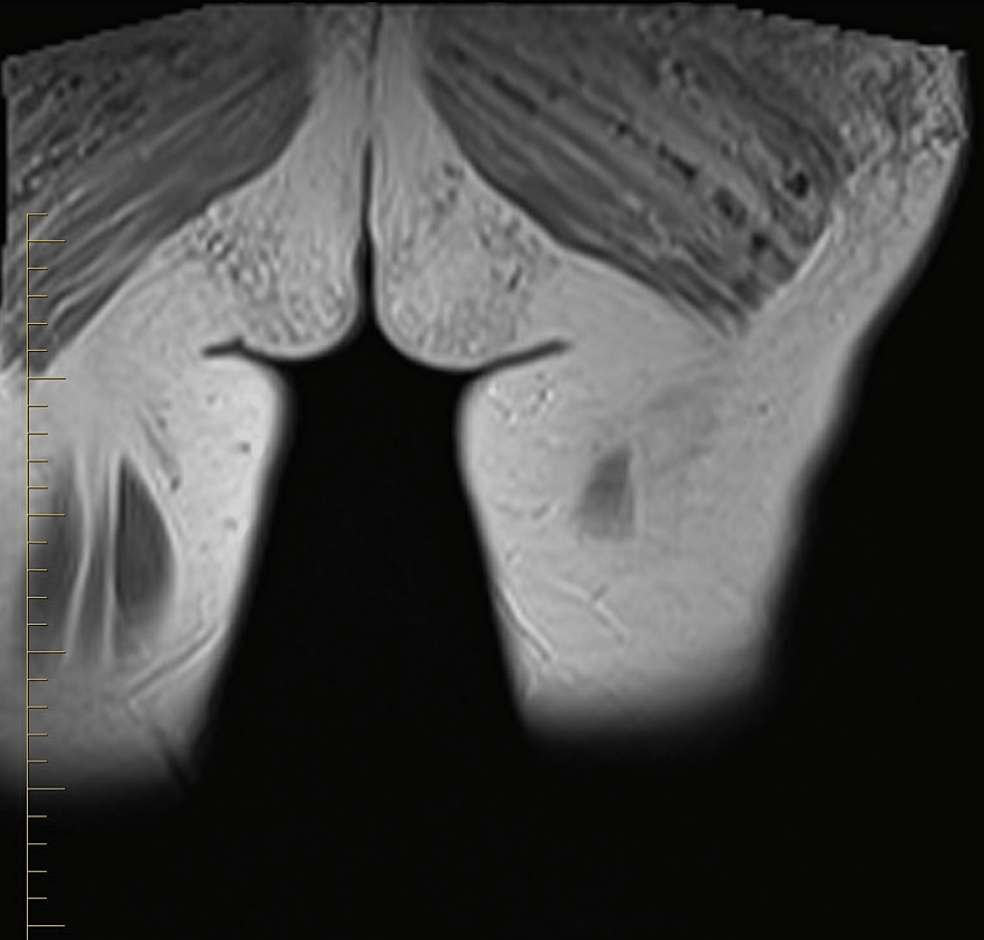
Coronal non-fat saturated T1 weighted image shows heterogenous signal intensity in the partially imaged right gluteus maximus muscle.

In this case, a 32-year-old female patient presented with persistent hip pain that developed six months after hip augmentation cosmetic surgery. The patient had no significant medical history, and laboratory tests were unremarkable. MRI revealed post-procedural changes from prior hip augmentation surgery. There are intramuscular cystic changes with heterogeneously signal intensity, as seen on axial T2 fat-saturated images (Figure 1). Intramuscular edema within the left gluteus maximus muscle is also seen on the axial T2 fat-saturated image (Figure 2). The axial T1 weighted fat-saturated image demonstrates cystic intramuscular changes (Figure 3). The coronal non-fat saturated T1 weighted shows similar signal intensity changes in the partially imaged right gluteus maximus muscle (Figure 4).

FBR is an inflammatory response to the presence of exogenous material in the body, including implants, prosthetics, and other foreign bodies. The mechanism underlying FBR involves the activation of immune cells, particularly macrophages, which attempt to phagocytize and remove the foreign material [3]. This process triggers the release of cytokines, growth factors, and other inflammatory mediators that can cause tissue damage, fibrosis, and chronic inflammation [4].

In the case presented, the FBR was likely triggered by the presence of the implant material (liquid silicone) used in hip augmentation surgery. The patient was referred back to the plastic surgery office for further management. The patient underwent treatment with 25 mg of etanercept, administered subcutaneously on a twice-weekly basis, following the confirmation of negative tuberculin (purified protein derivative) test results. Within two weeks, there was a significant reduction in pain and tenderness experienced by the patient.

In cases without a localized granuloma reaction or abscess formation, conservative management is done and a course of steroids, etanercept, or tetracycline antibiotics may be used as treatment options [4]. Surgical removal of the implant material is also an option for the treatment of FBR in more complex or advanced cases where there is localized granuloma or abscess formation. [1]. However, the decision to perform revision surgery should be carefully considered, as it carries its own risks, including infection, bleeding, nerve injury, and implant loosening [5].

This case report highlights the importance of early recognition of FBR in patients undergoing hip augmentation cosmetic surgery, with emphasis on the typical MRI features in these patients. The typical findings of scattered cystic areas on T2 weighted images and the increased intramuscular signal must raise suspicion for FBR in a patient with an appropriate history. MRI has an important role in assessing for additional complications, such as abscess or granuloma formation, and also in assessing the extent of the disease. Proper patient selection, use of appropriate surgical techniques, and preventive measures, such as avoiding implant materials that are known to trigger FBR, can minimize the risk of this complication.

## Conclusions

This case report emphasizes the significance of MRI in identifying FBR following hip augmentation cosmetic procedures. FBR is an uncommon yet severe consequence of such surgeries, which manifests as an inflammatory response to foreign materials within the body. Early detection is crucial to mitigate potential complications, and MRI serves as a vital diagnostic tool in this regard.

Although management of FBR can be challenging, conservative treatments are generally employed, while surgical intervention is reserved for more complex cases. It is essential for healthcare professionals to be cognizant of this potential complication and to ensure patients are adequately informed about the risks and benefits associated with the procedure prior to surgery.
